# Cutaneous T-cell Lymphoma of the Mycosis Fungoides Variant: A Case Report

**DOI:** 10.7759/cureus.112542

**Published:** 2026-07-12

**Authors:** Casandra Monserrat Dominguez Sánchez, Liz Soralla Hernandez Ramirez

**Affiliations:** 1 Internal Medicine, Instituto Mexicano del Seguro Social, Mexico City, MEX; 2 Dematology, Instituto Mexicano del Seguro Social, Mexico City, MEX

**Keywords:** biopsy, cutaneous t-cell lymphoma, granulomas, interferons, mycosis fungoides, non-hodgkin lymphoma, skin, skin neoplasms, t lymphocytes

## Abstract

Primary cutaneous lymphomas comprise a diverse group of T- and B-cell-related diseases. Their heterogeneity has made diagnosis and classification challenging. Of all the existing subtypes, mycosis fungoides is the most common. We present the case of a 62-year-old Mexican man who had been treated for chronic dermatitis and subsequently underwent a skin biopsy. The biopsy revealed mycosis fungoides, and he is currently being treated with corticosteroids while awaiting re-evaluation to determine if he requires phototherapy. Mycosis fungoides presents a diagnostic and therapeutic challenge among cutaneous lymphomas, characterized by an insidious onset and a multifactorial etiopathogenesis.

## Introduction

Mycosis fungoides is a primary cutaneous lymphoma that originates from T-cell and B-cell lineages. It often presents insidiously and may mimic numerous inflammatory dermatoses. Mycosis fungoides and Sézary syndrome occur in 53% of all cutaneous lymphomas and are classified as cutaneous T-cell lymphomas [1].

Cutaneous T-cell lymphomas are a rare disease. They represent only 4% of all non-Hodgkin lymphomas in the United States, with an incidence of approximately 6.4 to 7.7 cases per million people, according to data from the Surveillance, Epidemiology, and End Results Program. Within this group, mycosis fungoides and Sézary syndrome are the most frequently seen subtypes, and together they account for approximately two-thirds of all cutaneous T-cell lymphomas. It occurs more frequently in men than in women, in a ratio of 2:1, and is usually diagnosed around age 55, although the likelihood of developing it increases with age.

That said, neither mycosis fungoides nor Sézary syndrome is exclusive to older people: cases have also been documented in young adults and even children. Regarding prognosis, the role of age remains unclear. It has been observed that there are differences by ethnicity: the incidence is highest in non-Hispanic Black individuals (11.5 per million), followed by Hispanic individuals (7.9 per million) and Asian or Pacific Islander individuals [2].

In Mexico, the exact incidence is not well established due to its rarity and possible underdiagnosis. Primary cutaneous lymphomas have a low incidence (7-10 cases per 100,000) and are divided into those derived from T lymphocytes (70-85%) and from B lymphocytes (15-30%).

## Case presentation

**Figure 1:**
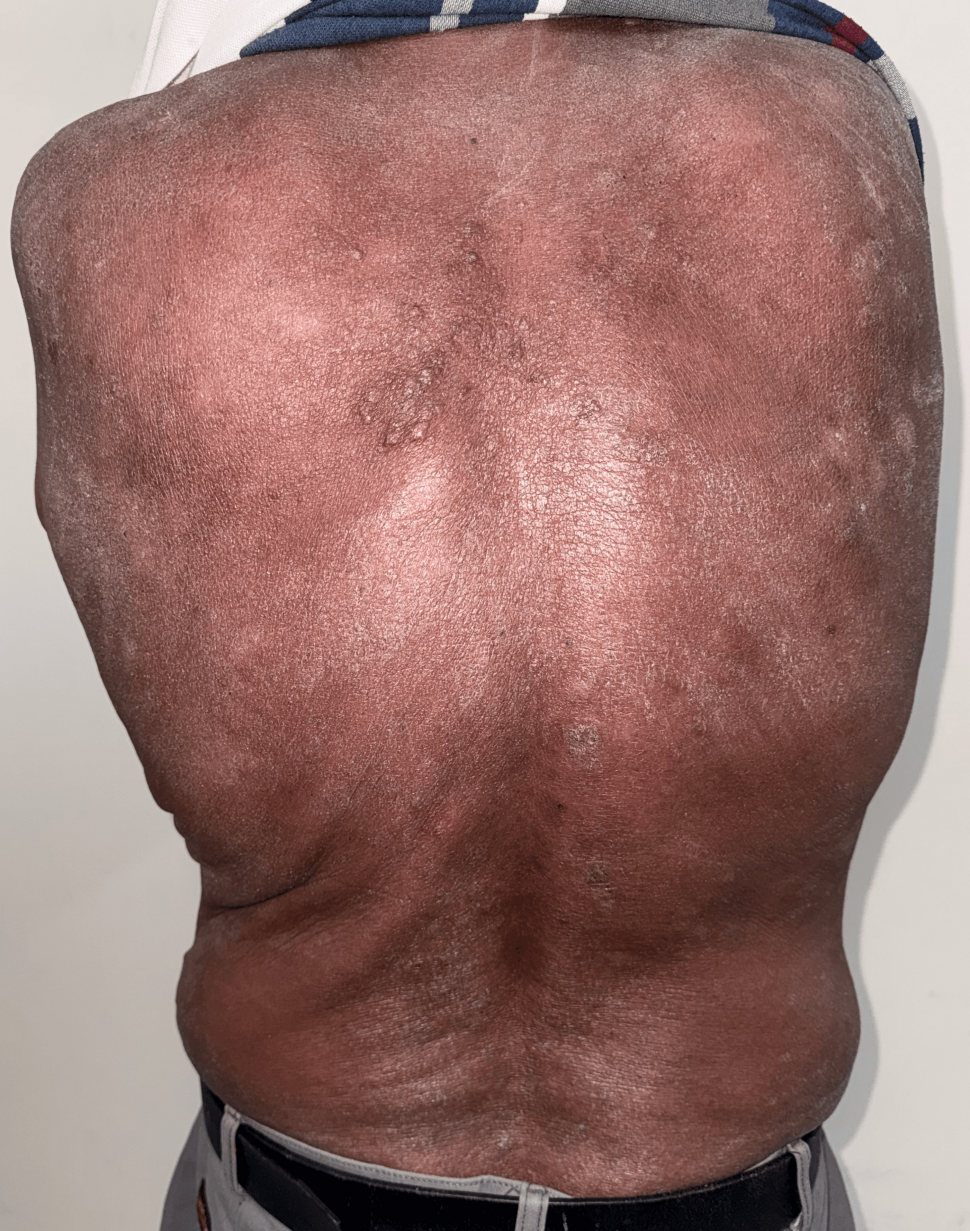
Mottled and reticulated dyschromia where patches of hyperpigmentation alternate with areas of hypopigmentation settled on a surface with evident skin atrophy

**Figure 2:**
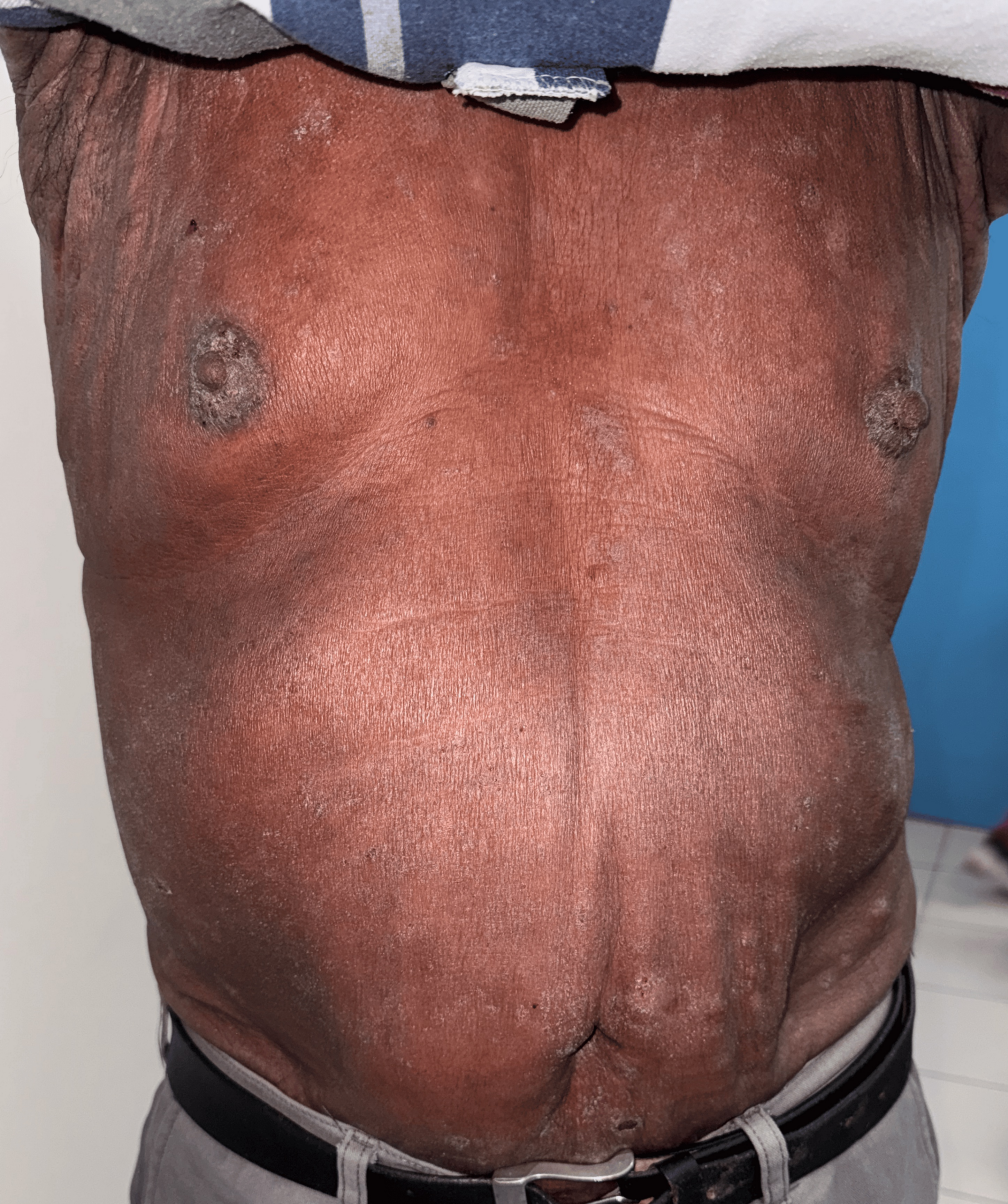
Diffuse and generalized dermatosis located on the anterior trunk with extension toward the axillary folds characterized by erythematous-brown hyperpigmentation

We present the case of a 62-year-old Mexican man, originally from Mexico City and residing in the State of Mexico, with a history of type 2 diabetes. Symptoms began at age 57 with thick, adherent, gray, erythematous, and scaly lesions affecting the face, forearms, and back, sparing the palms and soles. These lesions consisted of poikiloderma plaques and well-defined erythrosquamous scaly plaques with a rice paper appearance (Figures 1-2).

**Figure 3:**
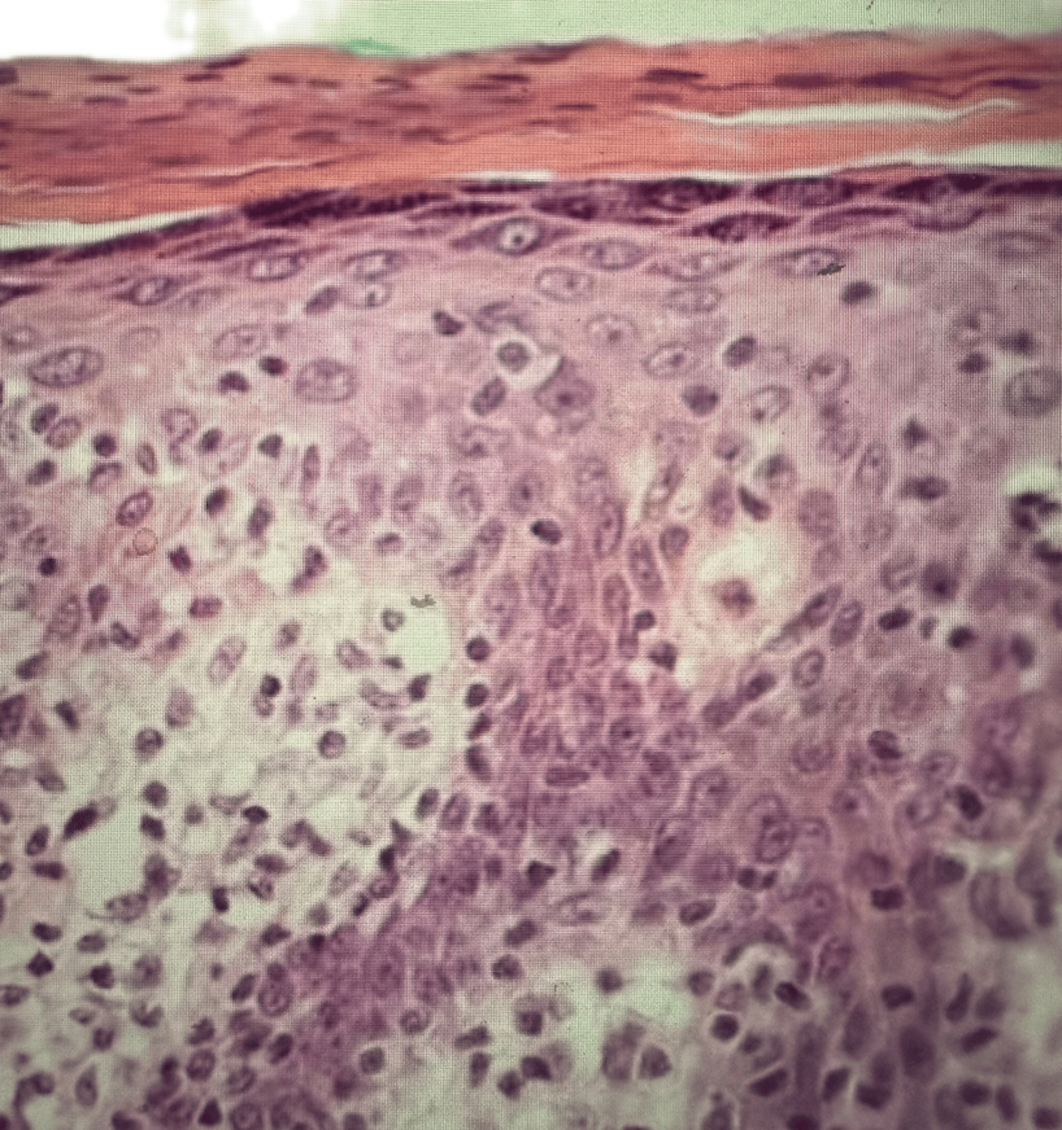
Active epidermotropism with basal alignment of atypical lymphocytes (10x) with hematoxylin and eosin staining

After being treated with topical steroids and emollients without apparent improvement, a biopsy was performed, which resulted in a diagnosis of mycosis fungoides (Figure 3).

He is currently receiving treatment in collaboration with the hematology service and is being treated with corticosteroids, awaiting further evaluation to determine whether he is a candidate for phototherapy.

## Discussion

Mycosis fungoides originates from a monoclonal proliferation of mature memory helper T lymphocytes with the CD4+/CD45RO+ phenotype. In its initial stages, the disease tends to remain confined to the skin for years, manifesting as erythematous macules with scaling or as infiltrated plaques that persist, generally in areas of the body not exposed to the sun.

In these early stages, mycosis fungoides does not usually affect overall survival. A good example of this phenomenon is the case of our patient, who has suffered from the disease for 15 years without it progressing to a more advanced clinical stage.

However, in approximately 20% of cases, the disease progresses. When this occurs, nodules or tumors may appear, solitary or multiple, and tend to ulcerate. It is common for these lesions to coexist with pre-existing macules or plaques, and in some cases, there may also be specific involvement of lymph nodes or visceral organs [3].

Diagnosis, staging, and treatment planning require a comprehensive approach, including a detailed medical history, a thorough and well-documented examination of the skin lesions, and histological studies that consider the immunological phenotype. In our patient's case, the available information is limited to the biopsy report [4].

Mycosis fungoides is clinically important because of its diagnostic complexity and therapeutic challenges. It is characterized by a gradual progression that hinders early diagnosis, as it mimics common dermatoses. Poor early diagnosis adversely affects prognosis and quality of life, which calls for an approach that accounts for the complexity of its etiopathogenesis. Classic mycosis fungoides manifests as patches and plaques on areas of the body not exposed to the sun, lesions that can slowly progress to tumors over time.

On the other hand, Sézary syndrome represents a considerably more aggressive variant with leukemic behavior. It is characterized by a characteristic triad: circulating neoplastic T-cells, erythroderma, and lymphadenopathy, although the latter is not always present.

It is worth highlighting the distinction between erythrodermic mycosis fungoides and Sézary syndrome, as they may appear clinically similar but have different origins. Erythrodermic mycosis fungoides is distinguished by the absence of circulating Sézary cells or by a very low count of them and is understood as a progression of mycosis fungoides proper. Sézary syndrome, in contrast, usually appears de novo, without a prior phase of mycosis fungoides [1].

The diagnostic process for patients with mycosis fungoides is often prolonged because the disease frequently mimics common inflammatory dermatoses. It frequently involves both clinical and histopathological misdiagnoses and ineffective treatments. It is not uncommon for the disease to be mistaken for an inflammatory dermatosis, such as psoriasis, in its early stages. When this occurs, the patient ends up receiving treatment with topical steroids or systemic immunomodulators for years, without a biopsy to support the diagnosis or adequate follow-up to detect the true progression of the disease.

To arrive at an accurate diagnosis, several steps cannot be omitted: a complete and detailed physical examination, identification of suspicious lesions, multiple biopsies from representative sites, and rigorous evaluation of the resulting pathological samples. Among the most important aspects of this process is establishing a precise correlation between clinical and histopathological findings, which is not always straightforward and sometimes requires validated scoring systems.

In this regard, the International Cutaneous Lymphoma Society developed a specific scoring system for the early diagnosis of mycosis fungoides that integrates clinical, histopathological, and immunohistochemical findings and also considers T-cell receptor gene rearrangement. This tool has proven useful precisely in cases where the clinical picture is not entirely clear from the outset [5].

Clinical variability necessitates individualized management, employing therapies ranging from cutaneous to systemic, with the choice based on disease stage and the presence of extracutaneous involvement. The treatment of mycosis fungoides requires essential multidisciplinary collaboration, in which dermatology is complemented by the expertise of hematologists in diagnosing and managing lymphoid neoplasms.

## Conclusions

Mycosis fungoides presents a diagnostic and therapeutic challenge among cutaneous lymphomas, characterized by an insidious onset and a multifactorial etiopathogenesis. While advances have been made in understanding and treating the disease, early diagnosis remains a challenge, and the need for curative or disease-modifying therapies persists. While skin-directed therapies have proven effective in managing most early cases of mycosis fungoides, an individualized approach is often needed due to the variability in disease progression.

First-line management involves skin-directed therapies such as topical corticosteroids and phototherapy, which, if unsuccessful, can be followed by systemic medications such as interferon-α, oral bexarotene, methotrexate, and novel antibody therapies; mycosis fungoides can also respond to localized radiotherapy, total skin electron beam therapy, and hematopoietic stem cell transplant.
